# Progress in Isoindolone Alkaloid Derivatives from Marine Microorganism: Pharmacology, Preparation, and Mechanism

**DOI:** 10.3390/md20060405

**Published:** 2022-06-20

**Authors:** Sijin Hang, Hui Chen, Wenhui Wu, Shiyi Wang, Yiwen Fang, Ruilong Sheng, Qidong Tu, Ruihua Guo

**Affiliations:** 1 College of Food Science and Technology, Shanghai Ocean University, Shanghai 201306, China; sjhang2022@163.com (S.H.); whwu@shou.edu.cn (W.W.); 2 Shanghai Engineering Center of Hadal Science and Technology, College of Marine Sciences, Shanghai Ocean University, Shanghai 201306, China; h-chen@shou.edu.cn; 3 AIEN Institute, Shanghai Ocean University, Shanghai 201306, China; may.canali@163.com; 4 Department of Chemistry, College of Science, Shantou University, Shantou 515063, China; ywfang@stu.edu.cn; 5 CQM-Centro de Química da Madeira, Campus da Penteada, Universidade da Madeira, 9000-390 Funchal, Portugal; ruilong.sheng@staff.uma.pt; 6 Jiangxi Provincial Key Laboratory of Drug Design and Evaluation, School of Pharmacy, Jiangxi Science & Technology Normal University, Nanchang 330013, China; 7 Shanghai Engineering Research Center of Aquatic-Product Processing & Preservation, Shanghai 201306, China; 8 Laboratory of Quality and Safety Risk Assessment for Aquatic Products on Storage and Preservation (Shanghai), Ministry of Agriculture, Shanghai 201306, China

**Keywords:** FGFC1, thrombus, fibrinolytic, *Stachybotrys longispora* FG216, *Stachybotrys microspora* IFO 30018

## Abstract

Compound **1** (SMTP-7, also FGFC1), an isoindolone alkaloid from marine fungi *Starchbotrys longispora* FG216 and fungi *Stachybotrys microspora* IFO 30018, possessed diverse bioactivities such as thrombolysis, anti-inflammatory and anti-oxidative properties, and so on. It may be widely used for the treatment of various diseases, including cerebral infarction, stroke, ischemia/reperfusion damage, acute kidney injury, etc. Especially in cerebral infarction, compound **1** could reduce hemorrhagic transformation along with thrombolytic therapy, as the traditional therapies are accompanied with bleeding risks. In the latest studies, compound **1** selectively inhibited the growth of NSCLC cells with EGFR mutation, thus demonstrating its excellent anti-cancer activity. Herein, we summarized pharmacological activities, preparation of staplabin congeners—especially compound **1**—and the mechanism of compound **1,** with potential therapeutic applications.

## 1. Introduction

The hemostatic system, consisting of the coagulation and fibrinolytic systems, is a vital physiological function in inhibiting hemorrhage and accelerating wound healing [1,2]. Fibrinolysis is regulated by plasminogen and activated by physiologic plasminogen activators: tissue-type plasminogen activator (t-PA) and urokinase-type plasminogen activator (u-PA). Meanwhile, the level of activated plasmin could be inhibited through the block of plasminogen activation by several specific molecules [3,4]. However, various clinical cases indicated that hereditary or acquired factors would enhance or weaken fibrinolytic systems, causing the disorder between the coagulation and fibrinolytic systems, which led to hemorrhage or thrombosis. Compared with a hemorrhage, thrombus formation develops much more gradually and imperceptibly, leading many patients’ deaths [5]. Current drugs on thrombosis include aspirin, ticlopidine, warfarin, and heparin; however, the risk of bleeding is a concern [6]. Therefore, small molecules, with different mechanisms of fibrinolysis action, are desired for new antithrombotics and thrombolytics.

Natural products, historically, possess various bioactivities for the treatment of human diseases [7,8,9,10,11,12]. Marine products, due to the unique marine environments, provide special structures of compounds differing from terrestrial ones. Up to now, over 28,175 chemical entities have been identified, with hundreds of new compounds discovered every year [13]. Numerous marine molecules have been approved for clinical treatments, such as anticancer cytarabine and analgesic ziconotide [14,15,16].

**Figure 1:**
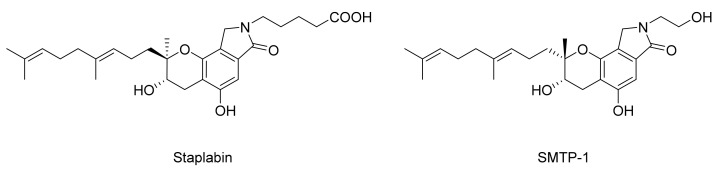
Structures of staplabin and SMTP-1.

**Figure 2:**
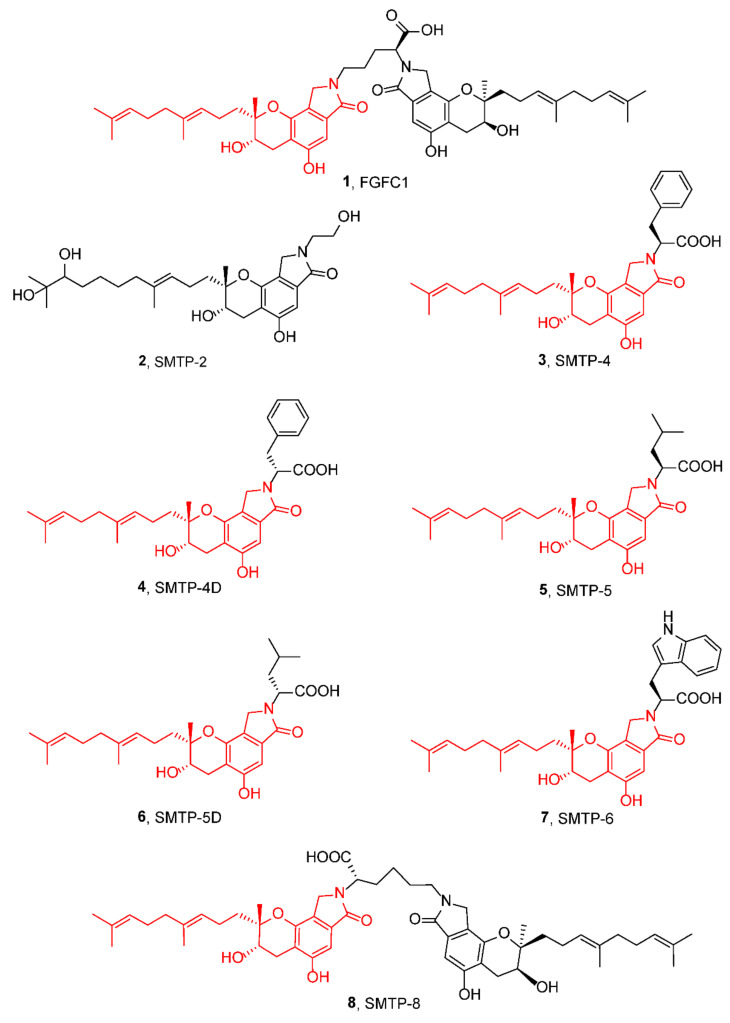

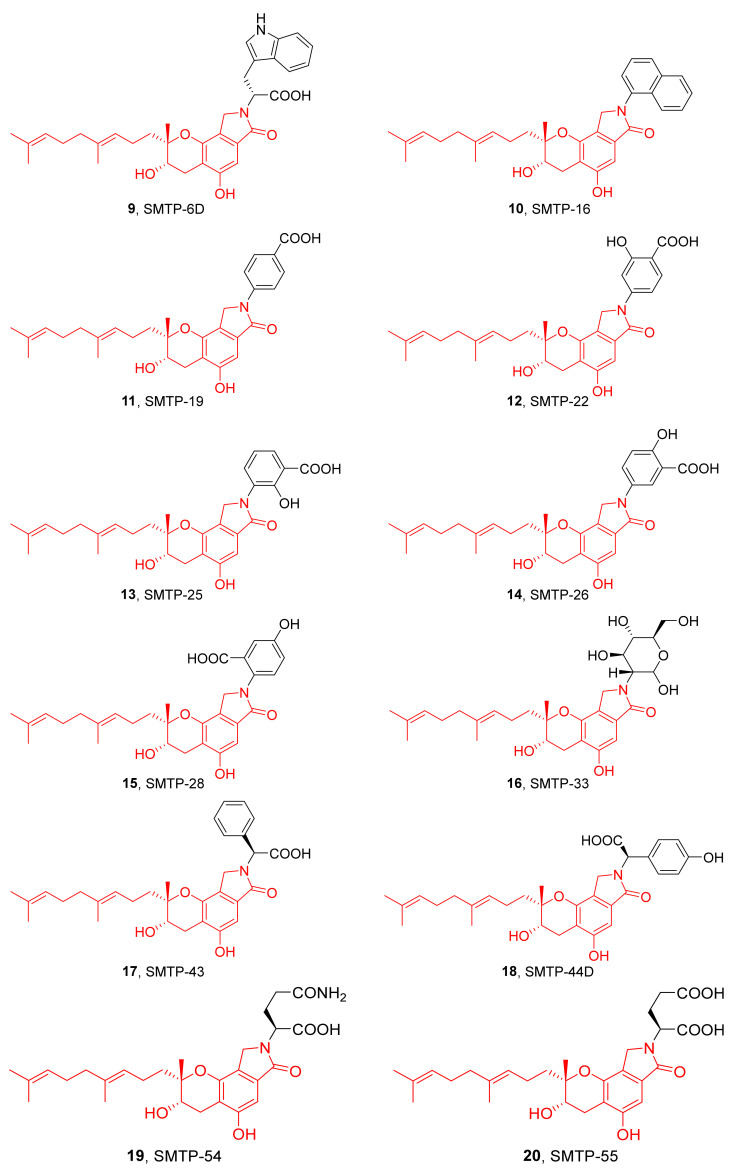

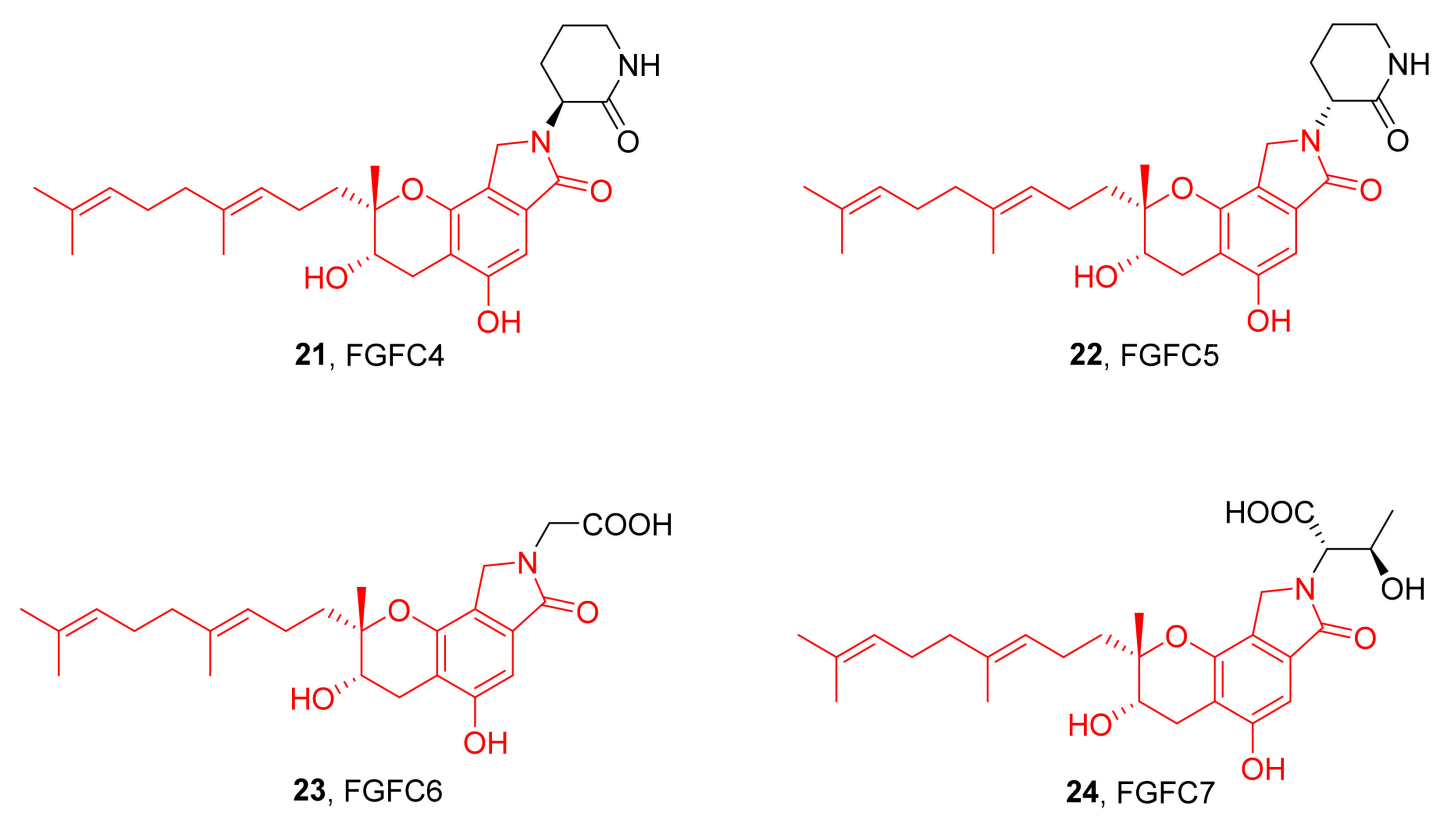
Structures of congeners and derivatives of staplabin.

In 1996, Kohyama et al. isolated SMTP-1 from fungi *Stachybotrys microspora* IFO 30018, with 20–30% higher plasminogen-fibrin binding action than staplabin, indicating the potential for thrombolysis therapy [17,18,19]. Staplabin, a triprenyl phenol, was the basic core of the SMTP family, and SMTP-1, as well as other congeners, were variants of staplabin (Figure 1) [17,20]. Then, a series of congeners, containing a tricyclic γ-lactam moiety, a geranylmethyl side-chain, and an N-linked side-chain, were isolated and showed plasminogen activation [21]. Compound **1** (Figure 2), with two staplabin cores bridged by ornithine, could increase urokinase-catalyzed plasminogen activation, fibrin binding of plasminogen, and fibrinolysis mediated by urokinase and plasminogen [20]. Meanwhile, it showed excellent clot clearance activity in vivo [22,23]. In a rat pulmonary embolism model, compound **1** (5 mg/kg) enhanced, by three-fold, the clot clearance rate above the spontaneous clearance group. Moreover, clot clearance of compound **1** was enhanced, further, in combination with u-PA [23].

At present, more than 60 congeners of staplabin have been isolated, which not only performed fibrinolysis activity but also exhibited various effects, such as anti-inflammatory, neuroprotection, and anti-cancer properties [1]. Other modified derivatives and structure–function relationships of compound **1** have also been studied. Scientists also confirmed the absolute configuration and preparation methods of congeners and derivatives, which shared similar absolute configuration (8S, 9S) and a staplabin core. Meanwhile, most monomeric analogues could be obtained by replacing the ornthine with non-basic amino acids or simple amines [24,25]. Herein, we focused on the diverse biological activities of compound **1**, which is a congener of staplabin (Figure 2).

## 2. Pharmacological Activity

### 2.1. Thrombolytic Activity

Thromboembolic disease is a main cause of mortality and disability. For instance, stroke is responsible for 5.2% of all mortalities in the world [26]. Most of them are ischemic strokes, which would trigger transient or permanent occlusion of cerebral vessels causing brain infarcts, cerebral tissue death, and focal neuronal damage after blocking for 6 h [27]. Therefore, the key to saving stroke patients is solving thromboembolism in an efficient way. It is needed to search for more potent and safer drugs for the inhibition and treatment of ischemic symptoms.

In 1999, Hu et al. isolated compound **1** from the fungus *S. microspora* IFO 30018, with a preliminary determination for its plasminogen activation and fibrinolysis activity at 80–150 μM in vitro [20]. In 2010, Hashimoto et al. established a novel cerebral infarction model for predicting cerebral infarction, in which generated embolus transferred to the brain in the right common carotid artery of Mongolian gerbils, induced by acetic acid [28]. In the same year, they assessed the therapeutic effect of compound **1** and t-PA in the cerebral infarction model [29]. The fibrinolytic activity of compound **1** (<20% of positive control) was lower than t-PA (>140% of positive control) in the 3 h after administration, but the activity of compound **1** increased about 3.5-fold during 1–3 h and was higher than t-PA after 3 h. It was attributed that the activity of compound **1** gradually increased. Meanwhile, compound **1** extended the therapeutic time window. Compared with a clear infarct in the cerebral hemisphere by t-PA (10 mg/kg) treatment, there was no visible infarction in the group of compound **1** at 3 h after ischemia. More importantly, there was little hemorrhagic region with 10 mg/kg of compound **1**, suggesting that it is a latent safe cerebral infarction therapy method. Hu et al., (2012) disclosed that compound **1** enhanced plasmin generation in vivo [20]. The level of plasmin-α2-antiplasmin complex, an indicator of plasmin formation, increased by 1.5-fold in male ICR mice after the treatment with 5 and 10 mg/kg of compound **1**. In 2014, the antithrombotic activity of compound **1** was further demonstrated in the male cynomolgus monkey model [30,31], which achieved excellent effects (Table 1). In addition, Ito et al. found that the combination therapy with warfarin and compound **1**, in the middle cerebral artery occlusion, improved the treatment safety and reduced hemorrhagic transformation [32]. Compound **1**, as a safe thrombolytic agent, relieved the side effects, such as severe infarction, edema, and hemorrhage, induced by warfarin in the middle cerebral artery occlusion model. All mice treated with compound **1** survived and the hemorrhagic severity score (1.3 ± 0.5) indicated decreased hemorrhagic transformation.

Then, Wang et al. isolated compound **1** from a rare marine fungus *Stachybotrys longispora* FG216 and evaluated its fibrinolysis activity [33]. Additionally, 0.1–0.4 mmol/L of compound **1** increased the Glu-plasminogen and Lys-plasminogen activation by 2.05–11.44 times in vitro. Meanwhile, 10 mg/kg compound **1** dissolved most pulmonary thrombus in the Wistar rat in vivo. Yan et al. further researched the thrombolysis and hemorrhagic activities of compound **1**, from *S. longispora* FG216, in vitro and on acute pulmonary embolism Wistar rat model in vivo [34]. Compound **1**, from 5 to 25 μM, induced fibrin hydrolysis in vitro; moreover, its thrombolytic activity was evaluated with fluorescence lung tissues in vivo. It was observed that compound **1**, of 5 and 10 mg/kg, displayed effective dissolving capacity (less fluorescence halo). Meanwhile, the euglobulin lysis time (ELT) was shortened for 30 s by the treatment of compound **1** in the Wistar rat model. Shortening ELT was related to the activation of the fibrinolytic system. Therefore, compound **1** exhibited fibrinolytic activity in vivo. Compound **1** (5, 10, and 25 mg/kg), especially, did not induce fibrinogenolysis at 30 min and 2 h after administration, which suggested that compound **1** reduced the risk of hemorrhage. Thus, compound **1** was a potential thrombolytic agent without hemorrhage [34]. In 2021, Gao et al. detected that compound **1**, with low concentration (0.096 mM), enhanced fibrinolytic activity by 2.2-fold in vitro; however, it inhibited fibrinolytic activity at excess doses (above 0.24 mM) [35].

Congeners **3**, **5**, and **7** enhanced fibrinolysis activities at 0.25 mM, in the ^125^I-Fibrin degradation experiment, by 2.3-fold, 1.9-fold, and 2.7-fold, respectively [36]. Congener **8** (80 μM) also increased fibrinolysis activity by eight-fold in the fibrin binding of ^125^I-plasminogen [20]. In 2003, Hu et al. isolated congeners **4**, **6,** and **9** with the activation effect on the urokinase-catalyzed plasminogen in vitro [24]. In 2012, congeners **11**–**13**, isolated from *S. microspore*, showed similar plasminogen activation activities when compared with compound **1** [37]. In 2018, Shibata et al. evaluated fibrinolysis activities of congeners **12** and **17** in an acetic acid-induced cerebral infarction mouse model [38]. Compared with compound **1**, of 10 mg/kg, congeners **12** and **17** reduced the size of the infarction area, neurological score, and edema percentage (Table 2).

To gain a deep insight into the antithrombotic effect, many studies attempted to illustrate the detailed mechanism for compound **1**. Hashimoto et al., firstly, confirmed excellent thrombolytic activity of compound **1** (no visible infarction area after treatment with 10 mg/kg for 3 and 6 h) in an acetic acid-induced novel embolic cerebral infarction model in vivo. They hypothesized that compound **1** could relieve cerebral infarction by combined effects, giving rise to studies on other activities of compound **1** [28]. In 2010, they demonstrated that compound **1** possessed thrombolytic and anti-inflammatory activities [29]. By the treatment with compound **1**, at 3 h after ischemia, mRNA expression of interleukin-1β (IL-1β), tumor necrosis factor-α (TNF-α), and interleukin-6 (IL-6) did not increase significantly. Therefore, compound **1** ameliorated hemorrhage and neurologic deficits, with a wide therapeutic time window in thrombolysis, by inhibiting inflammation. One year later, Akamatsu et al. further completed the anti-inflammatory mechanism of compound **1** in fibrinolysis [39]. Matrix metalloproteinase-9 (MMP-9) was significantly inhibited (92 kDa band) with compound **1** in transient focal cerebral ischemia, suggesting a cerebral neuroprotective effect of compound **1,** on ischemia/reperfusion injury, and a reduction risk for hemorrhagic transformation. Moreover, compound **1** inhibited the expression of an early superoxide anion and nitrotyrosine for 2 h after ischemia/reperfusion, so it showed anti-oxidative activity to reduce ischemia/reperfusion damage [39]. In 2014, Hanshimoto et al. observed that reactive oxygen species could cause overexpression of proinflammatory cytokines [40]. Compound **1** inhibited the overexpression of a signal transducer and activator of transcription 3, to extend the therapeutic time window in thrombolysis therapy, by exhibiting its anti-oxidative effect. In addition, Huang et al. observed the inhibitory activity of compound **1** on pro-MMP-9, which inhibited the degradation of the basal membrane and the blood–brain barrier, reducing the risk of hemorrhage [41]. Moreover, Koyanagi et al., (2014) found compound **1** performed better plasminogen activation activity with the presence of physiological cofactors [42]. Compound **1**, of 20–60 μmol/L, promoted the activation of Glu-plasminogen, by 10-fold, with phosphatidylcholine and phosphatidylserine. Meanwhile, compound **1** also showed promoted plasminogen activation activity, with the presence of gangliosides and oleic acid released from thrombus in the process of clot lysis. These endogenous cofactors might change the fifth kringle domain conformation of plasminogen to induce the interactions between compound **1** and the plasminogen, whose mechanism could be elucidated in detail in future.

Wu group also investigated the thrombolytic mechanism of compound **1**. Compound **1** of 0.1–0.4 mmol/L activated the Glu-plasminogen and Lys-plasminogen (2.05–11.44 folds), but it had no fibrinolytic activity in the absence of u-PA or a plasminogen in vitro [33]. Meanwhile, the treatment, with 10 mg/kg of compound **1** after 24 h, performed efficiently in the pulmonary embolism Wistar rat model, meaning that u-PA and plasminogen mediated its thrombolytic effect. Furthermore, Wu group detected enzymatic kinetic parameters of compound **1** by chromogenic-substrate associated with *p*-nitroaniline from the enzymatic reaction [43]. The results indicated that the increase in *k*_cat_ and *k*_cat_*/K*_m_ activity was related to the concentration of compound **1**, which exhibited 26.5-fold and 22.8-fold activity at 40 μg/mL. Moreover, the affinity of plasminogen and pro-uPA to the enzyme substrate presented a faint decrease with an increasing concentration of compound **1**, as the *K_m_* increased (from 0.413 to 0.484 μmol/L) along with the increasing concentration of compound **1** (0–40 μg/mL). The results further proved that reciprocal activation of pro-uPA and plasminogen was critical to the fibrinolysis activity of compound **1**, which enhanced the maximum catalytic efficiency and total catalytic activity of fibrinolysis. The fibrinolysis activity of compound **1** featured an enzymatic kinetic characteristic.

**Figure 3:**
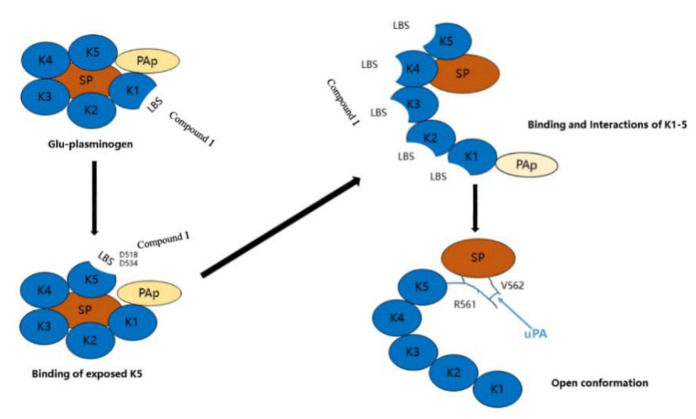
The mechanism of plasminogen activation by compound **1**.

**Figure 4:**
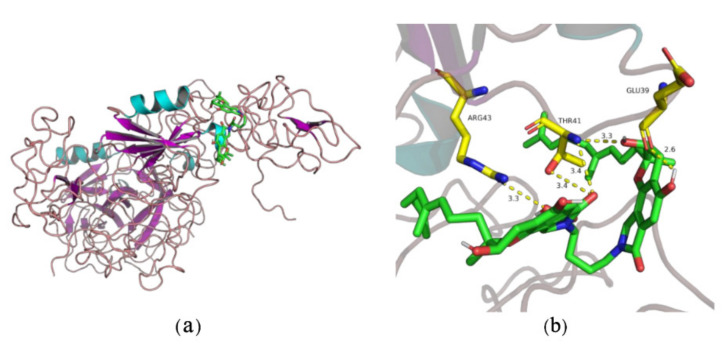
The interactions between compound **1** and plasminogen: (**a**) binding site; (**b**) the 3D docking model of compound **1** with plasminogen.

Wu group further studied the interaction mechanism between compound **1** and plasminogen [35]. The Glu-plasminogen, in the bloodstream, contains a Pan-apple domain (PAp), five kringle domains (KR1-KR5), and a serine protease domain (SP). Lysine-binding sites (LBS), in kringle domains of plasminogen, were essential for the interaction of plasminogen and compound **1** [44,45,46]. Compound **1**, firstly, bound the LBS of KR1, while KR1 activated and mediated the interaction between plasminogen and the C-terminal lysine moiety on fibrin. KR5 dropped from PAp and was exposed to closed plasminogen temporarily [36]. Then, compound **1** formed hydrogen bonds with Asp518 and Asp534 in KR5, which induced conformational change and structural rearrangement. After that, additional LBSs on kringle domains were exposed, leading the movement of PAp. Therefore, these additional LBSs interacted with compound **1**, causing an open conformation of plasminogen, which could be easily activated by u-PA (Figure 3). The theoretical binding mode showed that compound **1** formed a stable complex with Glu39, Thr41, and Arg43 in plasminogen with hydrogen bonds (Figure 4).

**Figure 5:**
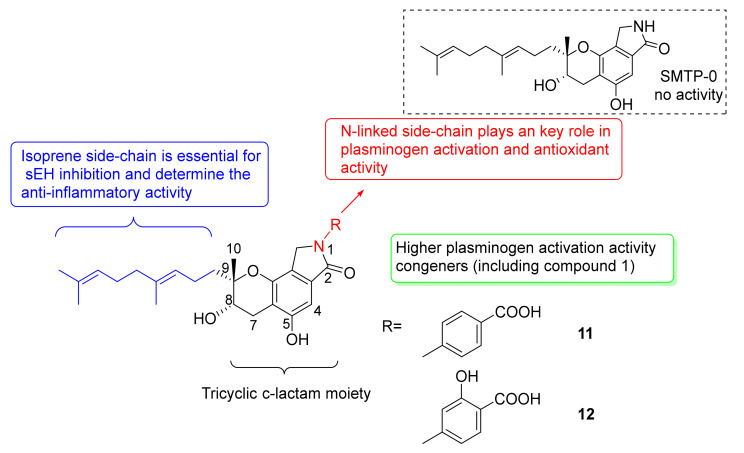
Structure–activity relationships of congeners **11** and **12**.

The structure–activity relationships of the plasminogen modulator compound **1** and its congeners have been studied in detail. Most congeners contain the same geranylmethyl side-chain, but they bear different *N*-linked side-chains. Congener **2** possessed a hydroxylated geranylmethyl side-chain [17] and could not enhance plasminogen binding to the activated plasminogen. Thus, the side-chain of geranylmethyl plays a key role in promoting plasminogen activation. In 2016, Otake et al. found that the geranylmethyl side-chain of congeners was critical to inhibitory activity of soluble expoxide hydrolase (sEH), an enzyme mediating anti-inflammatory action [47]. sEH lost inhibitory activity with the increasing number of hydroxyl groups on geranylmethyl side-chains or missing geranylmethyl side-chains, and the terminal hydroxy group of side-chain led to the damage of cellular localization. For the *N*-linked side-chain, Hasumi et al. isolated the simplest congener (SMTP-0) without an *N*-linked side-chain, which had no plasminogen activation effect [48] (Figure 5). It can be concluded that the *N*-linked side-chain was essential for plasminogen-modulating activity. In 2010, Hasegawa et al. confirmed the crucial role of the *N*-linked side chain in modulating plasminogen [21]. The congeners, without ionizable groups in *N*-linked side-chain, were inactive in plasminogen activation, such as with congener **10**. Moreover, the congeners, with an aromatic group and a negatively ionizable group on the side-chain, were more active for the enhancement of plasminogen activation than those with an aliphatic group and a negatively ionizable group, such as congener **3** (*E*_max_ = 15-fold). Koide et al. further isolated a series of SMTP congeners with different *N*-linked side-chains and evaluated their bioactivities. Congeners **11** (*E*_max_ = 126-fold) and **12** (*E*_max_ = 159-fold) were as potent as compound **1** (*E*_max_ = 102-fold) in plasminogen-modulating activity [37]. Only these congeners could express higher plasminogen-modulating and anti-oxidative activities, and other isomers or phenolic hydroxy groups, at different positions, did not present satisfactory activities in plasminogen activation. Moreover, the congeners with *N*-linked side-chains showed anti-oxidative activities too. All the congeners bearing a phenolic hydroxy group and a carboxylic acid group displayed higher anti-oxidative activities. Congener **12**, especially, possessed more than 1.7 times the anti-oxidative activity in comparison to compound **1** (Figure 5).

Pharmacokinetics is an essential evaluation system for the development of new drugs, which finally determines the metabolism and efficacy of drugs in vivo [49]. In 2013, Su et al. observed the pharmacokinetics and tissue distribution of compound **1** in Wistar rats [50]. Compound **1** had a half-life (*t*_1/2_) of ca. 22.37 min, and it was suitable for two-compartment models, by intravenous administration, for 10 and 20 mg/kg. From the viewpoint of tissue distribution, compound **1** was present in the highest concentration in the liver, but it had low or undetectable concentrations in the brain, suggesting that compound **1** did not cross the blood–brain barrier (the results were wrong, and compound **1** could cross the blood–brain barrier). In 2019, Ma et al. evaluated pharmacokinetic properties in beagle dogs and permeability characterization in Caco-2 cells [51]. *t*_1/2_ of compound **1** was determined in dogs’ brains, and *t*_1/2_, in beagle dogs, was about two times longer than in Wistar rats (48.7 min in average). Moreover, compound **1** performed low penetrability in a human Caco-2 cell’s monolayer model and the rapid distribution into organs, suggesting intravenous injection was more appropriate than oral. In addition, absorption and transportation characteristics of compound **1** had been studied [52]. In Caco-2 cells model, compound **1** expressed passive diffusion of the absorption pattern, and it was not the substrate of P-gp, indicating that compound **1** could cross the blood–brain barrier. Therefore, compound **1** had the potential to be a thrombolytic agent for the treatment of occluded cerebral vessels.

**Figure 6:**
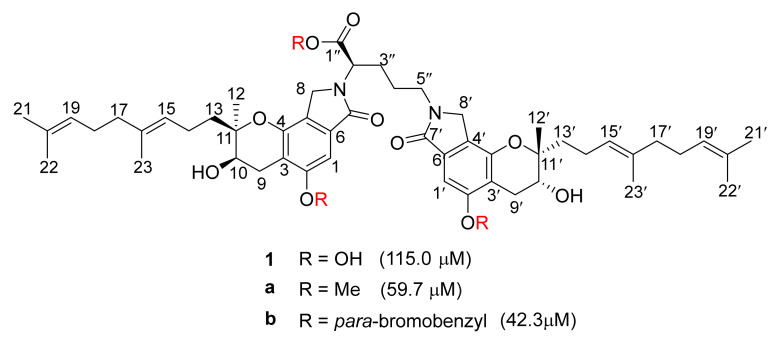
The modification of compound **1**.

The modification of compound **1** enhanced fibrinolytic activity to access more efficient and safer thrombolytic agents. In 2021, Wang et al. synthesized a series of compound **1** derivatives through the modification of phenyl groups, at the C2-OH and C2′-OH positions on compound **1**, and evaluated their fibrinolytic activities (Figure 6) [53] (The compound, modified by Wang et al., was the enantiomer of compound **1** (8S, 9S)). Derivative **a**, with methyl, and derivative **b,** with *para*-bromobenzyl, presented significant fibrinolytic activity with the EC_50_ values of 59.7 µM and 42.3 μM, respectively. Derivative **b** showed rapidly increasing fibrinolytic activity in the early stage (0–40 min), dose-dependently. Furthermore, derivative **b** displayed weak activities of inducing apoptosis and anti-inflammation on HeLa cells, suggesting that derivative **b** was a potential antithrombotic agent.

### 2.2. Effects on Inflammation and Oxidant Related Damage: In Reperfusion of Occluded Vessels

A large portion of tissue damage in diseases is caused by inflammation. In 2000, it had already been confirmed that inflammation could affect the coagulation system and regulation, which was responsible for thrombotic complications in vivo [54]. In 2005, it was found that patients with inflammatory diseases were more likely to develop thrombosis. For instance, the patients with inflammatory bowel disease suffered from a three-fold risk of pulmonary embolism or vein thrombosis [55]. In 2008, the unique role of inflammation was focused in the formation of venous thrombus [56]. Thus, researchers investigated the relationship between the reduced damage of ischemia/reperfusion and anti-inflammatory activity by the treatment of compound **1**.

Mammalian sEH contributes to inflammatory response through hydrolyzing lipid signaling molecules, and it has been developed as a potential therapeutic target [57,58]. More than 100 sEH inhibitor patents have been published for the treatment of diabetes, hypertension, pain, and cardiovascular diseases [59,60]. The geranylmethyl side-chain of compound **1** is crucial to the inhibitory activity of sEH [47]. Therefore, compound **1** and other staplabin congeners possessed great anti-inflammatory potential.

**Figure 7:**
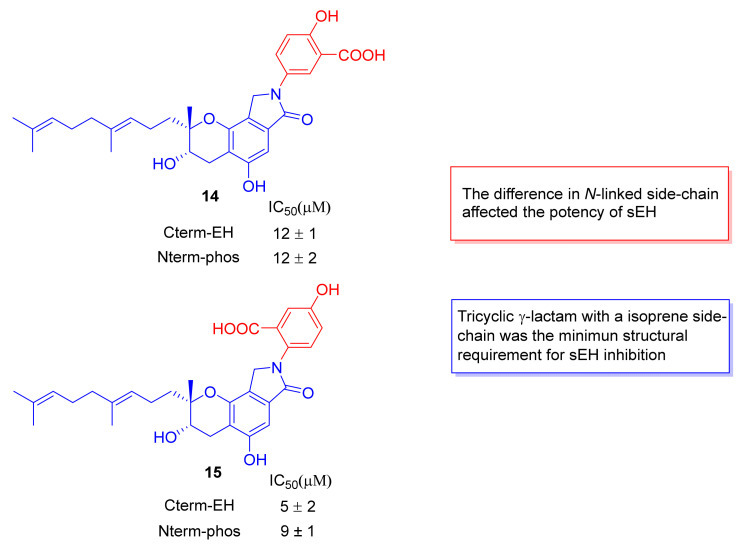
The structure of congeners **14**, **15,** and the SARs study of inhibitory effect on Cterm-EH and Nterm-phos.

sEH is a bifunctional enzyme with a C-terminal domain (Cterm-EH) and an N-terminal domain (Nterm-phos). Cterm-EH catalyzes hydrolysis of epoxyeicosatrienoic acids (EETs, an endogenous signaling molecule involved anti-inflammation), and Nterm-phos hydrolyzes lipid phosphates [60,61,62]. Therefore, the inhibition to Cterm-EH is the key to inhibit inflammation. Mastsumoto et al. performed sEH inhibition kinetic analysis of congeners [63]. Congeners **14** (IC_50_ = 12 ± 1) and **15** (IC_50_ = 5 ± 2) had better Cterm-EH inhibitory activities in comparison to congeners **10** (IC_50_ > 100) and **16** (IC_50_ > 100). Therefore, although the geranylmethyl side-chain was essential to the inhibitory activity, the nature of *N*-linked side chains also affected the inhibitory potency of sEH (Figure 7). Compound **1** inhibited the hydrolysis of EETs with IC_50_ of 6.5 μM. SMTP-0 (IC_50_ = 1.2 μM) and congener **18** (IC_50_ = 9.2 μM) were also efficient for inhibiting the hydrolysis of EETs [63]. Meanwhile, both 10 mg/kg of compound **1** and congener **18** improved neuritis symptoms in a rat Guillain–Barré syndrome model, and they alleviated symptoms of ulcerative colitis and Crohn’s disease in mice. The results proved that compound **1** and staplabin congeners possessed great anti-inflammatory potential [63].

**Figure 8:**
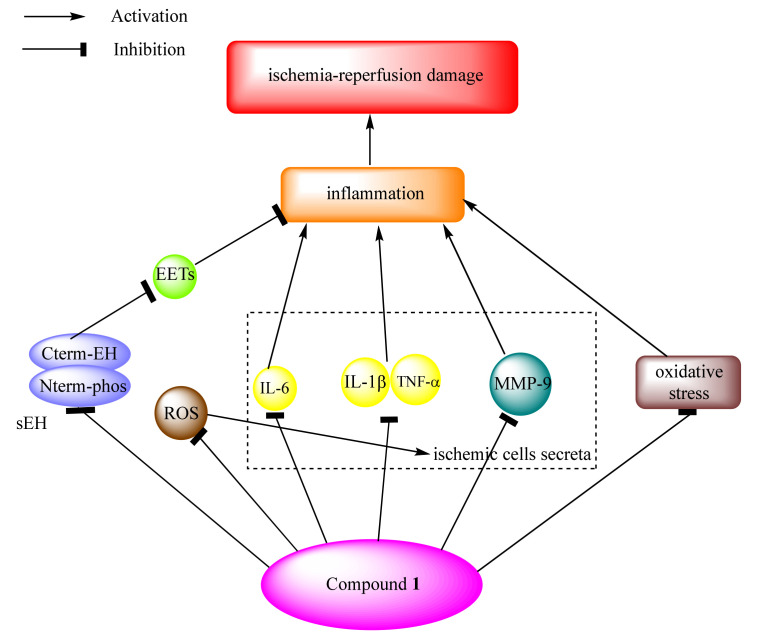
The anti-inflammatory and anti-oxidative mechanisms of compound **1** in ischemia–reperfusion damage.

Occluded vessels reperfusion could produce reactive oxygen species (ROS), which would stimulate ischemic cells, secreting excessive pro-inflammatory and inflammatory cytokines, such as IL-6, IL-1β, and TNF-α. The overexpressed cytokines cause damage, hemorrhage, and even inflammation in cerebral vessels, which is a major factor in ischemic brain injury [64]. Shibata et al. observed little hemorrhagic region with compound **1** (10 mg/kg), in the model of cerebral infarction mice, in comparison with 10 mg/kg t-PA treatment; moreover, they investigated the involved mechanism [29]. mRNA expression of IL-6, IL-1β, and TNF-α were not increased by the treatment of compound **1**, in comparison to t-PA treatment, at 3 h after ischemia. Hashimoto et al. found that compound **1** decreased expression of IL-6, the signal transducer and activator of transcription 3, S100 calcium binding protein A8, and MMP-9 by microarray and RT-PCR analysis [40]. Therefore, compound **1** inhibited the secretion of pro-inflammatory and inflammatory cytokines to improve the hemorrhage and ischemic brain injury. Meanwhile, Akamatsu et al. detected that superoxide anions (one ROS in cerebral ischemia) were observed by hydroethidine signals at 2 h after reperfusion [39]. Hydroethidine signal was reduced by the treatment of compound **1** in comparison to the vehicle group, meaning that compound **1** inhibited the production of ROS to decrease reperfusion damage. In addition, the treatment of compound **1** reduced the expression of nitrotyrosine and MMP-9, causing attenuated ischemic neuronal damage. Moreover, inflammatory tissue could release proteolytic enzymes of MMP-9, which is associated with blood–brain barrier breakdown and hemorrhagic complications in cerebral infarction [65]. Ito et al. indicated that compound **1** inhibited the activation of MMP-9 to protect the blood–brain barrier from destruction and hemorrhagic transformation (pro-MMP-9: 88.9 ± 34.2; MMP-9: 5.0 ± 1.6) in mice [32]. Besides, Huang et al. suggested that compound **1** could inhibit oxidative stress to reduce ischemia/reperfusion injury [41]. Compound **1** decreased the expression of 4-hydroxy-2-nonenal (4-NHE), 3-nitrotyrosine, and 8-hydroxy-2′-deoxyguanosine (8-OHdG) significantly, which provided therapeutic benefits for ischemic stroke. Therefore, compound **1** possessed anti-inflammatory and anti-oxidative activities in the reperfusion of occluded vessels (Figure 8).

### 2.3. Neuroprotective Activity

In 2011, Akamatsu et al. first confirmed the intrinsic neuroprotective effect of compound **1** [39]. In the photochemical-induced thrombotic occlusion model of cynomolgus monkeys, compound **1** of 10 mg/kg improved the neurologic deficit by 29% and cerebral hemorrhage by 51%, after treatment for 24 h, in comparison to the saline control group [29]. Then, Shibata et al. further explored the mechanism of reducing brain damage [66]. Compound **1** inhibited the expression of 4-NHE and neutrophil cytosolic factor 2 (Ncf2) after treatment for 1–3 h. Additionally, 4-NHE is an oxidized product of lipid peroxidation, Ncf2 can stimulate the NADPH oxidase complex to produce SOD, and their levels would increase in the infarction area [67]. Therefore, compound **1** reduced lipid peroxidation and the generation of SOD, in cerebral infarction, to possess neuroprotective activity [66]. Moreover, Ito et al. evaluated the activation of MMP-9 with compound **1** treatment in a mouse model. Compared with the control group, compound **1**, of 10 mg/kg, inhibited the expression of MMP-9, which could digest the endothelial basal lamina and open the blood–brain barrier, causing neuro-inflammation [32]. Compound **1** showed less basal membrane damage and functional breakdown of the blood–brain barrier. Therefore, compound **1** reduced neuronal damage by inhibiting MMP-9 expression. In 2018, Huang et al. investigated the anti-inflammatory and antiapoptosis mechanisms of compound **1** for neuroprotective effects [68]. Compound **1**, of 10 mg/kg, decreased the expression of NF-κB, TNF-α, and NLRP3-positive cells, which involved the alleviation of neuroinflammation. Meanwhile, compound **1** reduced the expression of cleaved-caspase-3, suggesting the inhibition in cell death progress. Therefore, compound **1** treatment demonstrated less necrosis of neurons and high neuroprotective activity in the peri-ischemic area. The results showed that the neuroprotective activity of compound **1** was attributed to the anti-oxidative, anti-inflammatory, and anti-apoptosis mechanisms in cerebral infarction.

**Figure 9:**
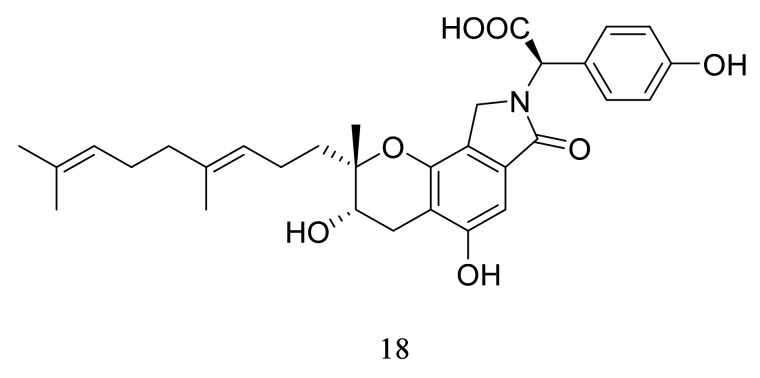
The structure of congener **18**.

In 2014, Matsumoto et al. found that congener **18** (10 mg/kg) (Figure 9) alleviated neuritis symptoms in a rat model presenting neuroprotective activity [63]. Shi et al. investigated the therapeutic effect of congener **18** in the neurovascular unit (NVU) and neurovascular trophic coupling damage [69]. Congener **18** ameliorated the NVU dissociation between pericyte, basal lamina, and astrocytic foot. It also improved the endothelial neuroprotective support for the outsider neurons. Moreover, congener **18** decreased the expression of TNF-α, 4-HNE, 8-OHdG, and cleaved caspase-3. Therefore, neuroprotective activity of congener **18** was due to its anti-inflammatory, anti-oxidative, and anti-apoptotic mechanisms. Congener **18**, especially, had therapeutic potential for diabetic neuropathy symptoms [70]. Congener **18** (30 mg/kg) improved the mechanical allodynia, thermal hyperalgesia symptoms, and neurological degeneration of DN in a streptozotocin-induced diabetes mouse model.

### 2.4. Effects on IgA Nephropathy and Acute Kidney Injury

IgA nephropathy (IgAN) has become a primary chronic glomerulonephritis worldwide, featuring mesangial cell proliferation and the deposition of IgA [71]. It causes a gradual decline in renal function, and 30% of patients will develop to the end stage of renal disease [72,73,74]. Kemmochi et al., (2012) investigated the therapeutic effects of compound **1** against IgAN [75]. In a mouse IgAN model, compound **1** (10 mg/kg) slightly reduced the deposition of IgA, but it had no effect on the serum concentration of IgA. The results suggested that compound **1** might inhibit the progression of IgAN through reducing the deposition of IgA in the glomerular mesangium. However, it was not effective in decreasing IgA production and treatment for terminal IgAN, indicating the limited therapeutic ability in IgAN.

Unlike IgAN, acute kidney injury (AKI) could cause rapid reduction in renal function [76]. The pathological condition of the kidney retained toxins and wastes, causing toxicosis and disorder in fluid, electrolyte, and acid–base balance [77]. It is estimated that 22% of hospitalized adults suffered from AKI [78]. Compound **1** showed less damage of ischemia-reperfusion in thrombolysis therapy. Meanwhile, ischemia-reperfusion played a major role in AKI renal damage. Therefore, in 2021, Shibata et al. studied the efficacy of compound **1** in renal damage [79]. Compound **1** improved the parameters of renal function (blood–urea nitrogen, creatinine levels in serum, creatinine clearance, and fractional excretion of sodium) and renal tubule damage. The therapeutic effect of compound **1** was derived from anti-inflammatory and anti-oxidative activities. The inhibition to sEH elevated the EET level, which inhibited tubular dysfunction and inflammatory factors, such as NF-κB, TNF-α, IL-6, and IL-1β. ROS production was also reduced after compound **1** treatment. Thus, the suppression of peroxidation led to less renal cell injury.

### 2.5. Effects on Cancer: Non-Small Cell Lung Cancer

The essence of cancer is the abnormal proliferation and differentiation of cells, which are dependent on angiogenesis [80]. Therefore, anti-cancer agents could identify and inhibit angiogenesis, thus representing an approach for cancer therapy [81]. Many patents on angiogenesis inhibitors have been published, such as angiostatin, endostatin, and thrombospondin. Therein, angiostatin is a hidden fragment of plasminogen with great antiangiogenic properties [82]. Congener **7** reduced vascular formation, along with proliferation and migration, to inhibit tumor growth and possessed plasminogen activation activity, causing a conformational change of plasminogen to dissolve thrombus [83]. Ohyama et al., (2004) reported that congener **7** also promoted the autoproteolytic of plasmin, inducing extensive fragmentation of the catalytic domain [83]. After urokinased-catalyzed plasminogen was activated by congener **7**, the catalytic domain of plasmin (activated plasminogen) rapidly degraded into 68–77 kDa fragments. These fragments blocked proliferation, migration, and vascular formation of endothelial cells, at concentrations of 0.3–10 μg/mL, meaning they provide potential applications of congener **7** for cancer treatment.

As the most common lung cancer, non-small cell lung cancer (NSCLC) accounts for approximately 80–85% of lung cancer diseases [84]. The clinical drugs for treating NSCLC are the epidermal growth factor receptor (EGFR) and EGFR-targeted tyrosine kinase inhibitors (TKIs). However, more than 80% patients gradually showed drug resistance after about 1 year of treatment with EGFR-TKI. Thus, it is necessary to discover new anti-tumor agents for treating NSCLC [85]. In 2022, Yan et al. observed the effects of compound **1** on erlotinib-resistant NSCLC and explored the underlying mechanism [86]. NSCLC cells were sensitive to compound **1** with IC_50_ = 7.45 ± 0.57 μM in vitro, especially for erlotinib-resistant NSCLC H1975 cells (IC_50_ = 9.22 ± 0.84 μM); meanwhile, compound **1** was relatively safe for normal cells. The accumulation of cleaved-PARP, cleaved-caspase-3, Bax, and the reduction in Bcl-2 revealed that compound **1** induced the cell apoptosis of NSCLC cells [86]. Then, they discovered the underlying mechanism of treating erlotinib-resistant NSCLC. Compound **1** induced mitochondria-mediated apoptosis, leading to increased ROS and reduced GSH. Thus, compound **1** caused apoptosis of erlotinib-resistant NSCLC cells [86,87]. In addition, compound **1** also inhibited the PI3K/Akt signaling pathway and the EGFR/PI3K/Akt/mTOR pathway. The abnormal activation of the PI3K/Akt pathway could cause TKI resistance, as well as invasiveness and migration of NSCLC [85]. Moreover, compound **1** showed high binding affinity to EGFR^T790M/L858R^ in molecular modeling, meaning compound **1** selectively exhibited anticancer activity on erlotinib-resistant NSCLC cells. Finally, compound **1** (10 mg/kg) had consistent anti-cancer effects in nude mice, meaning that it showed potential for erlotinib-resistant NSCLC therapy. In 2022, Feng et al. further observed that compound **1** downregulated the levels of CD4K and Cyclin D1 to arrest the cell cycle of PC9 cells at the G0/G1 phase [88]. Compound **1**, especially, inhibited the viability and proliferation of PC9 cells through the inhibition of the NF-κB signaling pathway. The results indicated that compound **1** had excellent anti-cancer activity on *EGFR*-mutant NSCLC cells, but it had weak or no effect on wild-type EGFR cells. It can be concluded that compound **1** might depend on the EGFR status to induce apoptosis of NSCLC cells.

## 3. Preparation of Compound 1 and Staplabin Congeners

Hu et al. isolated SMTP congeners from *S. microspora* in 2001 [25]. They found that the use of amino acids and amino alcohols significantly increased the production of congeners, and the obtained products were related to the type of added amino acid. The production of compound **1** and congeners **3**, **5**, and **7** increased by 7 to 45-fold, with the addition of Orn, Phe, Leu, Trp, and Lys at 100 mg/mL, which acted as precursors in culture. Therefore, the addition of precursors was an important procedure in the preparation of compound **1** and staplabin congeners.

**Figure 10:**
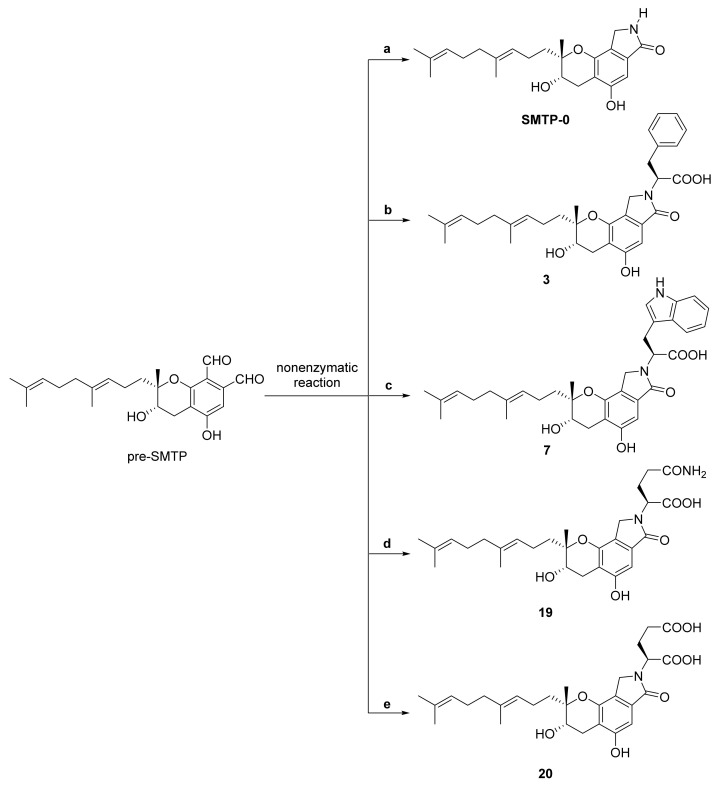
Synthesis of SMTP-0, as well as congeners **3**, **7**, **19,** and **20,** based on pre-SMTP (phthalaldehydes reaction). Pre-SMTP (100 μg/mL in acetone) was incubated with (**a**) 5 mg/mL ammonium acetate in acetic acid (1.5%, *v/v*); (**b**) 5 mg/mL *L*-phenylalanine in acetic acid (1.5%, *v/v*); (**c**) 5 mg/mL *L*-tryptophan in acetic acid (1.5%, *v/v*); (**d**) 5 mg/mL *L*-glutamine in acetone-water-acetic acid (50:50:1); (**e**) 5 mg/mL *L*-glutamic acid in acetone-water-acetic acid (50:50:1).

In 2012, Nishimura et al. isolated a new compound designated pre-SMTP from fungus *S. microspora*, which directly afforded SMTP congeners by reacting nonenzymatically (phthalaldehydes reaction) [89,90]. Pre-SMTP accumulated, with limited amounts of amine, in medium with *S. microspora*, and it consumed rapidly after increased amine feeding. Meanwhile, SMTP-0, as well as congeners **3**, **7**, **19,** and **20** were afforded, by reaction of pre-SMTP, with ammonium chloride, *L*-phenylalanine, *L*-tryptophan, L-glutamine, and L-glutamic acid, respectively. Thus, it is available to synthesize a variety of congeners, with different *N*-linked side-chain structures, through nonenzymatic reaction between pre-SMTP and an amine (Figure 10).

**Figure 11:**
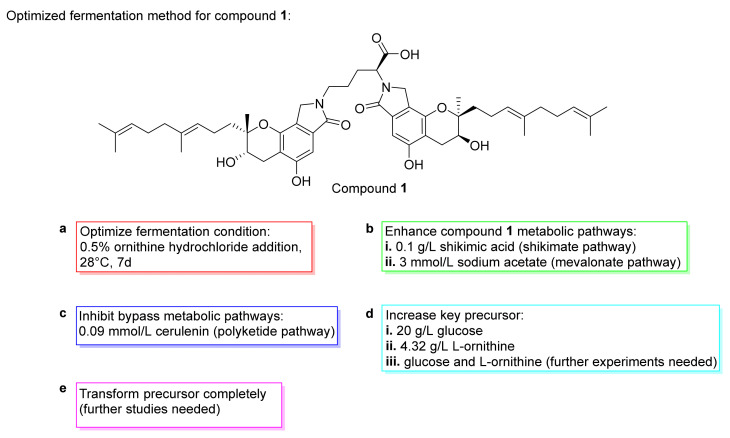
The optimized method for a compound **1** culture. On fermentaion basal medium, inducers (or precursor), and conditions: (**a**) 0.5% ornithine hydochloride, 28 °C, 7 d, 1.98 g/L; (**b**) (i) 0.1 g/L shikimic acid, yield increased by 10.4%; (ii) 3 mmol/L sodium acetate, yield increased; (**c**) 0.09 mmol/L cerulenin, yield increased by 14.6%; (**d**) (i) 20g/L glucose, 82.2 g/L; (ii) 4.32 g/L, L-ornithine, 95.9 g/L; (iii) and (**e**) need further research.

In 2013, Su et al. investigated the fermentation conditions of compound **1** isolated from *S. longispora* FG216 [91]. The results showed that the optimized fermentation conditions were as follows: 0.5% ornithine hydrochloride addition, 28 °C culture temperature, and 7 d (Figure 11a). The yield of compound **1** increased up to 1.98 g/L. In 2015, Wang et al. designed a metabolic regulation strategy to improve the production of compound **1** [92]. The results indicated that the carbon skeleton of compound **1** was synthesized through the shikimate and mevalonate pathways. Therefore, the addition of precursor shikimic acid and precursor sodium acetate increased the yield of compound **1** by 10.4%–14.6% (Figure 11b,c). Glucose and ornithine were the essential skeleton and structural core of compound **1**, respectively, which involved the synthesis of compound **1**. Along with 20 g/L glucose and 4.32 g/L *L*-ornithine provision, compound **1** increased up to 82.2 and 95.9 g/L (Figure 11d). During the fermentation, a transformation was observed from ornithine, FGFC3, and FGFC2 into compound **1**. Further research is needed for promoting transformation from ornithine to compound **1**, which will be able to increase production substantially (Figure 11e).

**Figure 12:**
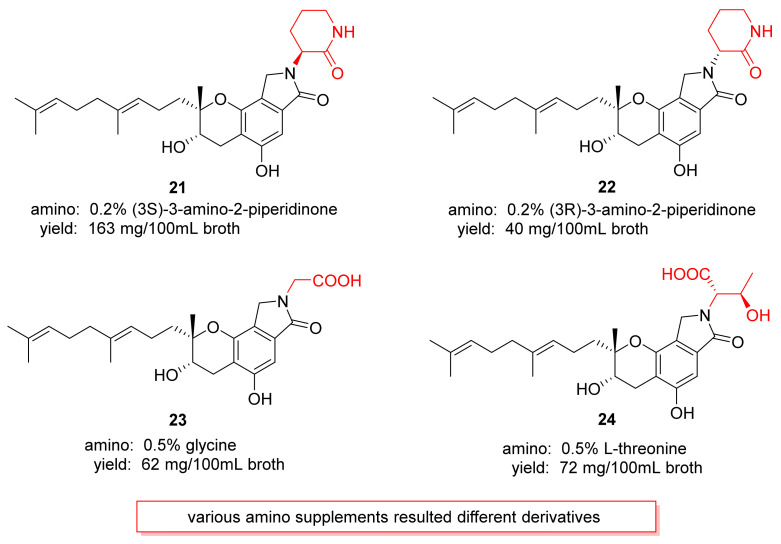
The structures of derivatives **21**–**24**.

Yin et al., (2017) studied the biosynthesis pathway in *S. longispora* FG216 [93]. The results were that three reported core genes and the nitrate reductase (NR) gene copy were the isoindolinone biosynthetic gene cluster in *S. longispora* FG216. NR is the rate-limiting enzyme of nitrate reduction. Therefore, nitrate reductase possibly played a role in the balance of ammonium ion concentration. Moreover, four new derivatives, **21**–**24**, were obtained by various amino supplements in *S. longispora* FG216 (Figure 12).

## 4. Conclusions

Compound **1** possesses rich bioactivities, such as excellent fibrinolytic, anti-inflammatory, and anti-oxidative activities. Compared with other anti-thrombotic agents, such as warfarin, compound **1** exhibits less hemorrhage risk for the treatment of thrombosis because it changes the conformation of plasminogen, in the presence of uPA, to activate the plasminogen. Moreover, the inhibition to sEH reduces inflammatory response, causing neuroprotection and less damage from the reperfusion of occluded vessels. The structure–activity relationships of compound **1** indicate that the *N*-linked side chain determines plasminogen activation activity, and the geranylmethyl side chain is essential for anti-inflammatory activity. Recent studies show that compound **1** possesses, surprisingly, anti-cancer activity toward EGFR-TKI-resistant NSCLC cells. Furthermore, the satisfactory pharmacokinetic property and optimized culture methods show that compound **1** has potential as a promising agent. Some modifications of compound **1** and staplabin congeners perform better in fibrinolytic or neuroprotective activity, thereby illustrating the high therapeutic potential.

## Figures and Tables

**Table 1:** The antithrombotic effect of compound **1** in the severe embolic stroke monkey model.

The Antithrombotic Effects	Efficacy (10 mg/kg)
consistent clot clearance	43.3 ± 40.5%
thrombotic middle cerebral artery occlusion recanalization	32.5-fold
neurologic deficit amelioration	29%
cerebral infarct reduction	46%
cerebral hemorrhage decrease	51%
infarct, edema and clot sizes reduction	65%, 37%, and 55%, respectively

**Table 2:** The fibrinolysis activities of compound **1**, as well as congeners **12** and **17**.

Fibrinolysis Activities (10 mg/kg)	Infarction Area Size Reduction	Neurological Score Reduction	Edema Percentage Reduction
Compound **1**	4.9 ± 1.1%	1.7 ± 0.4%	5.8 ± 1.0%
Congener **12**	4.4 ± 0.5%	1.7 ± 0.4%	4.6 ± 1.0%
Congener **17**	5.7 ± 1.2%	1.5 ± 0.5%	3.3 ± 1.4%

## Data Availability

Not applicable.
